# Eosinophilic pleural effusion as a manifestation of taeniasis

**DOI:** 10.4322/acr.2020.195

**Published:** 2020-11-20

**Authors:** Synrang Batngen Warjri, Tony Ete, Habung Mobing, Narang Naku, Vanlalmawsawmdawngliana Fanai, Shakeel Ahamad Khan, Arun Kumar, Animesh Mishra

**Affiliations:** 1 North Eastern Indira Gandhi Regional Institute of Health and Medical Sciences, Department of Cardiology, Supercare, Shillong, India; 2 North Eastern Indira Gandhi Regional Institute of Health and Medical Sciences, Department of Medicine, Shillong, India; 3 North Eastern Indira Gandhi Regional Institute of Health and Medical Sciences, Department of Surgery, Shillong, India

**Keywords:** Ascariasis, eosinophilia, pleural effusion, albendazole

## DEAR EDITOR

Herein, we describe a case of eosinophilic pleural effusion (EPE) associated with intestinal taeniasis. This diagnosis that demands a high index of suspicion and awareness and should be included in the differential diagnosis of EPE in the developing countries where parasitosis is highly prevalent.

A 21-year-old male patient presented with a history of low-grade fever, non-productive cough, and generalized weakness of one-month duration. On examination, he was conscious, oriented, and afebrile with the blood pressure of 120/80 mm of Hg, a pulse rate of 68 beats per min, and a respiratory rate of 20 per minute. There was no pallor, clubbing, icterus, or any enlarged lymph nodes. On the chest examination, respiratory sounds were absent on the bilateral infra-scapular and infra-axillary regions, where a stony dullness was achieved on percussion. Cardiac and neurological examination revealed no significant abnormalities. His abdomen was soft, with no organomegaly. Bowel sounds were normal on auscultation. Laboratory workup revealed a total leukocyte count of 38,500 cells/mL (reference range [RR]; 4000-11000 cells/ml) with a differential count showing 76% eosinophils, 14% neutrophils and 8% lymphocytes. Liver, thyroid, and renal function tests were within normal limits. p-ANCA (perinuclear anti-neutrophil cytoplasmic antibody), c-ANCA (cytoplasmic anti-neutrophil cytoplasmic antibody), rheumatoid factor and ANA (anti-nuclear antibodies) were all negative. Erythrocyte sedimentation rate (ESR) was 25 mm (RR; 0-22 mm/hr) after the first hour. Urinalysis was normal.

**Figure 1:**
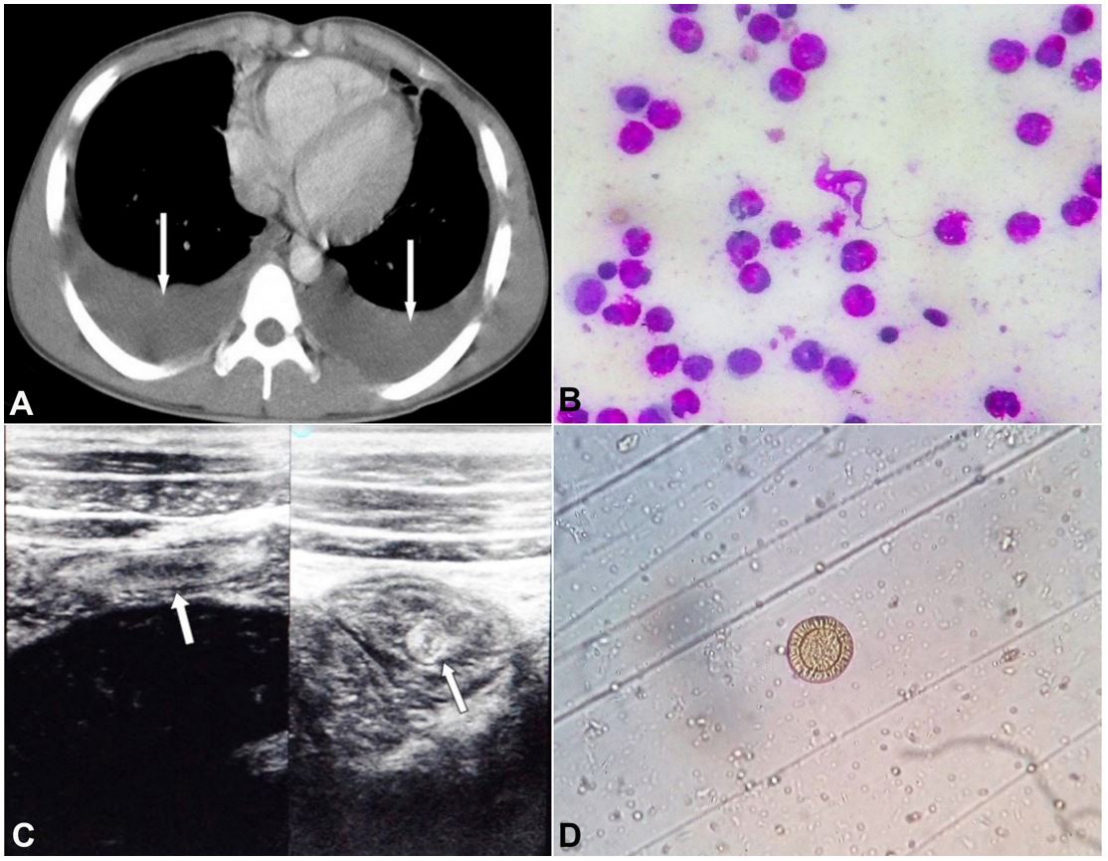
**A –** Thoracic CECT showing bilateral pleural effusion (white arrows); **B –** Pleural fluid smear - showing highly cellular fluid with a predominant presence of eosinophils; **C –** Abdominal ultrasonography showing the intestinal worm infestation (white arrows); **D –** Stool microscopic examination showing ova of Teania sp.

Bone marrow examination revealed a hypercellular marrow with increased eosinophilic cells (33%). No atypical or neoplastic cells were seen in the bone marrow study. Chest roentgenogram showed bilateral pleural effusions confirmed by the thoracic contrast-enhanced computed tomography (Figure 1A). The lung parenchyma appeared normal, and there were no signs of pulmonary tuberculosis, malignancy, pneumonia, or parasitic infections. Analysis of the pleural fluid showed an exudative fluid with high protein content (5.9 gm/dl) with a total cell count of 6200/mL, 84% of which were eosinophils (Figure 1B, Table 1). The ADA (adenosine deaminase) determination on the pleural fluid level was normal (39 U/L). Sputum examinations for acid-fast bacillus (AFB) and parasitic eggs were also negative. Abdominal ultrasonography revealed intestinal worm infestation (Figure 1C), and stool examination showed the presence of ova of *Taenia sp* (Figure 1D).

**Table 1:** Pleural fluid characteristics

	value
Protein	5.9 g/dl
Albumin	3.1 g/dl
Cell count	6200 cells/mL
Neutrophils	02%
Lymphocytes	14%
Eosinophils	84%
adenosine deaminase (ADA)	39 U/L

The patient was treated with 400 mg tablet of albendazole twice daily for two weeks, following which he reported the expulsion of worm segments in his stool. He was also started on daily doses of 20mg oral tablets of prednisolone, which was continued for 4 weeks. One week after the expulsion of the worm, the patient reported relief of symptoms, and his total leukocyte count returned to normal. His eosinophilia had also resolved by the first week. A repeat bone marrow aspiration also showed a normal marrow study. Pleural effusion, however, took about 4 weeks to resolve completely. Subsequent follow up revealed no recurrence of pleural effusion or eosinophilia.

The pulmonary manifestations of parasitic infections in humans are diverse and include transient pulmonary infiltrates, focal lung cysts, pneumothorax, consolidations, eosinophilic pneumonia, and pleural effusions. In 1932, the Swiss doctor Wilhelm Löffler described a syndrome of acute transient respiratory illness associated with blood eosinophilia and radiographic shadowing. This was attributed to the pulmonary migration of the larvae of *Ascaris lumbricoides*.^1^ Pleural effusions as a manifestation of parasitic infestations had also been described in paragonimiasis, hydatidosis, and amoebiasis.^2^

Eosinophilic pleural effusion (EPE) is characterized by an eosinophil count of ≥ 10% in the pleural fluid. It has been estimated to occur in 5-16% of all pleural effusions.^3^ The etiologies of EPEs are varied and include malignancies, bacterial, viral or fungi infections, parasites, pulmonary embolism, drug reactions, asbestos exposure, or the presence of air or blood in the pleural space.^3^ ^,^ ^4^ EPEs associated with parasitic infections are most commonly described in paragonimiasis.^2^ ^,^ ^5^ EPEs have also been reported in association with other parasitic infections such as toxocariasis, echinococcosis, and giardiasis.^6^ ^-^ ^8^

The pathogenesis of pulmonary eosinophilia due to parasitic infections is uncertain. Parasites such as *Ascaris lumbricoides*, *Necator americanus*, *Ancylostoma duodenale,* and *Strongyloides stercoralis*, whose life cycle in the human host includes the passage through the lung, can possibly induce an eosinophilic immune response leading to pulmonary eosinophilia. Other parasites such as toxocara, echinococcus, and paragonimus can penetrate the intestinal wall and spread to various tissues (including the lung), leading to local and systemic allergic and inflammatory responses. The life cycle of both *Taenia saginata* (beef tapeworm) and *Taenia solium* (pork tapeworm) in the human host does not involve migration through the lung. However, the *Taenia solium* eggs can penetrate the intestinal wall and develop cysticerci in any organ, typically in muscles, subcutaneous tissues, brain, and eyes.

The precise mechanism of EPEs in intestinal taeniasis remains speculative and most likely results from increased production and migration of eosinophils from the bone marrow into the pleural space in response to secretion of Interleukin (IL)-5 and Interleukin-4 by T-helper2 (Th2) lymphocytes. This response is similar to a Th2-type hypersensitivity reaction leading to tissue eosinophilia in allergic diseases. EPEs due to parasitic infestations also have higher levels of thymus and activation-regulated chemokine (TCAR), which is a selective chemoattractant for Th2 cells.^5^ TCAR stimulates the accumulation of Th2 cells into the pleural fluid leading to increase levels of IL-5 and IL-4, which in turn induce eosinophilic migration into the pleural cavity. These observations suggest that EPEs due to parasitic infections are a Th2-mediated process.

Even taking into account this background of uncertain pathogenesis of EPEs, we believe that the association of the *Taenia sp* infestation was related to the EPE, in our patient. A retrospective study by Wang et al.^9^ described a practical approach to diagnosing pleural parasitic infestations based on the presence of respiratory symptoms, pleural effusions, parasite or parasite eggs in the stool, EPEs and parasite-specific antibodies in the blood. By Wang’s recommendations, a definitive diagnosis of EPEs due to parasitic infestation can be made in our patient. Also, the resolution of the effusion, blood eosinophilia, and leukocytosis following deworming and expulsion of the parasite support the diagnosis of intestinal helminthiasis as the causative agent. This patient remained asymptomatic with no recurrence of the effusion or blood eosinophilia over 30 months of follow up. Follow up stool examination during this period did not reveal any evidence of intestinal helminthic re-infection. Similar cases of pleural effusions resolving after the treatment of the associated parasitic paragonimus or giardia infection have also been reported.^8^

The initial diagnostic workup of patients with EPEs should be directed towards identifying the likely responsible etiology. Malignancies are the most common causes of EPEs, and a thorough search for any neoplastic disease is always warranted. Proper drug history is essential to rule out drug-induced reactions. Drugs that have been implicated in EPEs include dantrolene, nitrofurantoin, bromocriptine, methotrexate, methysergide, amiodarone, bleomycin, procarbazine, procainamide, isotretinoin, valproic acid and fluoxetine.^10^ A thorough investigation should also be done to rule out conditions such as Churg-Strauss Syndrome, Hyper-eosinophilic Syndrome, and Tropical Pulmonary Eosinophilia. In developing countries such as ours, it is also essential to rule out tuberculosis as a cause of EPEs. In addition to all these various etiologies, a physician should always consider parasitic infections in the differential diagnosis of EPEs, especially in tropical and developing countries where parasitic infections are endemic.
